# Direct observation of pure pentavalent uranium in U_2_O_5_ thin films by high resolution photoemission spectroscopy

**DOI:** 10.1038/s41598-018-26594-z

**Published:** 2018-05-29

**Authors:** T. Gouder, R. Eloirdi, R. Caciuffo

**Affiliations:** European Commission, Joint Research Centre, Directorate for Nuclear Safety and Security, Postfach 2340, DE-76215 Karlsruhe, Germany

## Abstract

Thin films of the elusive intermediate uranium oxide U_2_O_5_ have been prepared by exposing UO_3_ precursor multilayers to atomic hydrogen. Electron photoemission spectra measured about the uranium 4*f* core-level doublet contain sharp satellites separated by 7.9(1) eV from the 4*f* main lines, whilst satellites characteristics of the U(IV) and U(VI) oxidation states, expected respectively at 6.9(1) and 9.7(1) eV from the main 4*f* lines, are absent. This shows that uranium ions in the films are in a pure pentavalent oxidation state, in contrast to previous investigations of binary oxides claiming that U(V) occurs only as a metastable intermediate state coexisting with U(IV) and U(VI) species. The ratio between the 5*f* valence band and 4*f* core-level uranium photoemission intensities decreases by about 50% from UO_2_ to U_2_O_5_, which is consistent with the 5*f* ^2^ (UO_2_) and 5*f* ^1^ (U_2_O_5_) electronic configurations of the initial state. Our studies conclusively establish the stability of uranium pentoxide.

## Introduction

Uranium oxides play an important technological role as nuclear fuel for electricity production^1,2^. Despite decades of extensive investigations, much remains to be discovered about the peculiarity of their structural, chemical and physical properties^3–9^, or about the subtleties of the surface chemistry mechanisms governing the interactions between uranium oxides and the environment^10–13^. Progress on the latter issue is essential to make much needed advances in all aspects of treating waste from the nuclear fuel cycle. Furthermore, uranium oxides have been studied as catalysts^14–16^ and in thermal and photolytic hydrogen production^17^.

In solid oxides, uranium exists in three oxidation states (IV, V and VI), usually associated with different crystallographic structures. U^IV^O_2_, the most commonly encountered oxide and the most widely used commercial nuclear fuel, can easily incorporate oxygen in its cubic fluorite structure, and a number of distinct crystallographic phases have been identified in the stoichiometric range U^IV^O_2_-U^VI^O_3_^18–23^. Initially, the extra oxygen ions in UO_2+x_ (with x up to about 0.2) are accommodated in interstitial positions of the original fluorite structure^19^. A further increase of the oxygen content is accompanied by a distortion of the crystal structure and by the formation of complex oxygen clusters^24,25^. For an O/U ratio larger than 2.25 one first observes a transition from cubic to tetragonal symmetry^26,27^ then, for oxides higher than U_3_O_7_ and close to U_2_O_5_, a transformation from the fluorite-type to a layered structure similar to the one of U_3_O_8_^25,27–33^.

The chemical properties of the oxides vary strongly with the oxidation state of uranium. Water solubility of uranium-oxide-based nuclear waste increases by 6 orders of magnitude from U(IV) to U(VI)^34^, so that the oxidation during storage of the initial UO_2+x_ is an important safety issue^35^. Therefore, redox processes on uranium oxides have been the subject of intense research. In particular, to what extent oxygen incorporation into UO_2_ directly oxidizes U(IV) into U(VI) and to what extent the intermediate U(V) if being formed, has been a matter of extensive debate. It has been suggested that fission products, such as Ce or Y, stabilize the U(V) state and thereby inhibit corrosion and dissolution^36,37^.

Although pentavalent uranium can be stabilized in uranyl complexes and can exist in aqueous solution^38–40^, the occurrence of U(V) in the solid state is uncommon. It is well established in thermodynamically stable ternary systems, for instance in CrUO_4_ and FeUO_4_^41^ or in KUO_3_ and NaUO_3_^42^, but in binary oxides its presence has only been reported as a metastable intermediate state coexisting with U(IV) and U(VI) species^43^. Direct evidence for the presence of U(V) in binary oxides has been provided by high energy resolution x-ray absorption spectroscopy measurements at the uranium *M*_*4,5*_ absorption edges (3d_3/2,5/2_)^44^. These experiments demonstrate that the conversion of UO_2_ in U_3_O_8_ progresses through the three oxidation states, U(IV)-U(V)-U(VI), as predicted by electronic structure calculations^45^, with U(IV) and U(V) species present in U_4_O_9_ and U(V) and U(VI) contained in U_3_O_8_.

The pure pentavalent uranium oxide U_2_O_5_ was first identified by Rundle *et al*.^27^ in 1948 with an orthorhombic layered structure representing an oxygen-deficient variant of U_3_O_8_. Allotropes with a monoclinic fluorite-type structure and a hexagonal layered structure have been reported later as the result of a thermal treatment at high temperature (673–1073 K) and high pressure (30–60 kbar) of a mixture of UO_2_ and U_3_O_8_^28,29^. However, the existence of U_2_O_5_ as a stable compound at ambient temperature and pressure conditions has been questioned for a while and a lower limit of x = 0.56–0.6 has been suggested for the single-phase region below U_3_O_8_^23,24^.

The stability of U_2_O_5_ has been recently investigated by electronic structure calculations. Density functional theory (DFT) simulations based on the Local Density Approximation including the on-site Coulomb interaction U (LDA + U) suggest that the orthorhombic form of U_2_O_5_ (δ-U_2_O_5_) is not thermodynamically stable^45^. On the other hand, using the Perdew-Burke-Ernzerhof exchange-correlation functional with on-site Coulomb correlations (PBE + U) within the Generalized Gradient Approximation (GGA), Brincat *et al*.^46^ predict a stable δ-U_2_O_5_ structure containing exclusively U(V) ions in mixture of distorted octahedral and pentagonal bipyramidal coordination sites. A similar approach has been used by Molinari *et al*.^47^ to compare the relative stability of various candidate structures for U_2_O_5_. These authors conclude that the most stable U_2_O_5_ structure is the Np_2_O_5_-type monoclinic one (containing uranyl square and pentagonal bipyramids linked by edge-sharing into sheets), whereas δ-U_2_O_5_ would become energetically favoured only at high temperatures or pressure. The difficulty of preparing single-phase samples of U_2_O_5_ is reflected in the paucity of experimental data, so that no information is available on the physical properties of this important oxide.

In corrosion experiments, U(V) has been observed by photoemission spectroscopy (PE) while exposing a UO_2_ surface to oxidizing conditions (radiolytic oxidants, oxygen, anodic potential)^48^. Pentavalent uranium can be easily identified from the energy of the shake-up satellite around the characteristic U*4f* doublet. Such a satellite, associated with intrinsic energy loss processes, appears in PE spectra as a sharp peak at a binding energy that depends on the uranium oxidation state. However, a common feature of corrosion experiments is a large gradient of oxidation states from the surface (exposed to the oxidants) and the bulk of the sample, because slow diffusion of the oxidant prevents the system from reaching equilibrium. This may produce mixed valence, as an artefact of incomplete reaction. This situation can be overcome by using films thin enough (some tens of atomic layers deposited on an inert substrate) to obtain a complete diffusion.

Here, we report the growth of homogeneous, single-valence U_2_O_5_ thin films of 30 monolayers thickness, obtained by mild reduction of a UO_3_ precursor multilayer in a hydrogen atmosphere. High resolution x-ray photoelectron spectroscopy provides direct and quantitative evidence for a uranium 5*f* ^1^ electronic configuration, as expected for the U(V) oxidation state. U_2_O_5_ is very sensitive to reduction: even short sputtering reduces it to UO_2_, so it is hard to observe on conventionally sputter-cleaned surfaces. Additionally, because U_2_O_5_ occurs in a region with easily modifiable oxygen composition, most bulk studies simply missed it and saw instead mixed valence compounds such as U_3_O_8_ (U(V)/U(VI) mixture) or U_4_O_9_ (U(IV)/(V) mixture).

## Results and Discussion

UO_2_ is by far the best characterized of the uranium oxides and a large number of experimental spectroscopy studies have been reported^49^. The U*4f* emission is characterized by a spin-orbit splitting of about 10.9(1) eV and by a satellite peak located at 6.9(1) eV above the primary lines^50,51^. The satellite has an inter-atomic origin and has been attributed to charge transfer or shakeup processes^52^. The energy separation between satellite and main emission line depends on the energy difference between the extended, occupied O*2p* states and the localized unoccupied U*5f* states. This is important, because the U*5f* states can be expected to move up in energy with the oxidation state of uranium, while the O*2p* states stays at an approximately constant energy. Final states effects are expected, because electrons are transferred from the 2*p* bonding orbital of the ligand to the open 5*f* or 6*d* uranium shells. Although a detailed theoretical description of the mechanism leading to the formation of the satellite is missing, it is empirically known that the U*4f* satellite energy is a marker of the uranium oxidation state in oxides. In the case of compounds containing U^VI^ ions, for instance, satellites appear at about 4 and 10 eV above the primary line, with intensities of less than 10% of the main peaks, whereas pentavalent uranium in non-binary compounds is revealed by one satellite in the interval from 7.8 to 8.3 eV^51^. It is also known that the degree of covalency in the metal-ligand bond affects the energy of the satellites, which becomes smaller in less-ionic compounds^53^.

**Figure 1:**
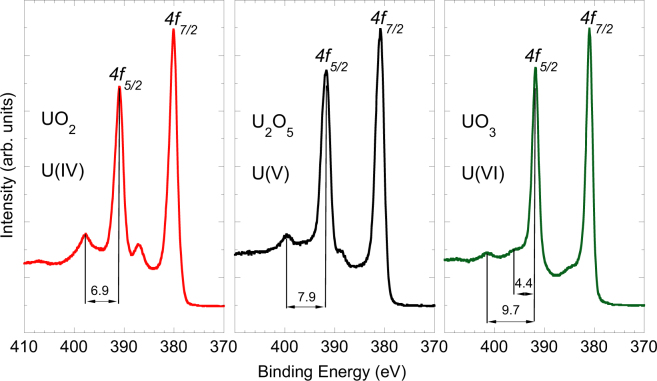
Uranium 4*f* core level X-ray Photoemission Spectra recorded for U(IV) in UO_2_ (left panel), U(V) in U_2_O_5_ (central panel), and U(VI) in UO_3_ (right panel). Data have been collected on thin films of about 20 monolayers thickness in ultra-high vacuum. The relative energy between the satellite peak and the 4*f*_5/2_ (4*f*_7/2_) emission line is used as a marker for the oxidation state of the uranium atoms.

The U*4f* core level photoemission spectra obtained in this study for UO_2_, U_2_O_5_, and UO_3_ thin films of about 30 monolayers (ML) thickness are shown in Fig. 1. The U*4f* XPS observed for UO_2_ exhibits narrow and symmetrical pointed peaks (FWHM = 2.09(5) eV), with maxima at 380.2(1) and 390.9(1) eV for the 7/2 and 5/2 components of the spin orbit split doublet. These values, together with the satellite energy at 6.9(1) eV above the main line, indicate a UO ratio corresponding to stoichiometric UO_2_.

The UO_3_ films has been produced by exposing a UO_2_ film to atomic oxygen at 573 K. As shown in Fig. 1, symmetrical sharp lines (FWHM = 1.67(3) eV) are observed, with a spin orbit splitting of 10.8(1) eV and two satellites peaks at 4.4(1) and 9.7(1) eV from U*4f*_5/2_, which are characteristic for the oxidation state U(VI)^51^. About the 4*f*_7/2_ peak, only the 4.4(1) eV satellite is visible as the ~10 eV one is hidden by the much stronger U*4f*_*5/2*_ peak. See the supplementary file for the peak fitting of UO_3_, U_2_O_5_ and UO_2_ spectra.

Intermediate oxides have been produced by exposing homogeneous UO_3_ films (about 20–30 monolayers thickness) to atomic hydrogen. The exposure was done at 673 K to ensure an atom mobility sufficient for obtaining homogeneous materials. The adopted procedure is milder than oxidation of UO_2_ by atomic oxygen, which at saturation dose always ended up as UO_3_. Moreover, sputtering artefacts can be excluded because the H plasma is not energetic enough to eject lattice oxygen atoms by physical knock-off and the momentum transferred by the hydrogen atoms to the much heavier oxygen atoms is small.

It turned out that reduction by atomic hydrogen does not proceed down to UO_2_ but stops at U(V). The U4*f* XPS shown in the central panel of Fig. 1 presents main peaks slightly broader (FWHM = 2.18(4) eV) than those observed for UO_2_ and UO_3_. They are separated by a spin-orbit splitting of 10.8(1) eV, with the U*4f*_7/2_ lying at 380.9(1) eV binding energy. Both U*4f* lines have a satellite peak located at 7.9(1) eV higher binding energy. This value is intermediate between those observed in UO_2_ and UO_3_, and lies in the range (7.8–8.3 eV) reported for a variety of compounds containing U(V)^51^. We therefore assume that the satellite indicates the presence of the U(V) oxidation state in a binary U-O system. The absence of spectral features at 6.9(1) and 9.7(1) eV above the 4*f* doublet lines implies that the compound is monovalent and corresponds to U_2_O_5_.

Uranium oxide bulk samples previously reported always appeared as mixtures containing UO_2_ or UO_3_, or in intermediate/mixed valence state with coexistence of U(V) with U(IV) or U(VI), as for U_4_O_9_ and for U_3_O_8_ respectively. Here we show that a pure sample of U_2_O_5_ can be prepared *in-situ*. The observation of U_2_O_5_ was claimed by Teterin *et al*.^54^ upon leaching of U_3_O_8_ in sulphuric acid followed by a thermal treatment in He atmosphere. However, while in our study only one main peak (*4f*_*5/2*_ and *4f*_*7/2*_) is observed in the U*4f* XPS, in agreement with the spectra reported for KUO_3_^42^, a two-peak structure appears in the XPS spectra given in ref.^54^, suggesting the presence of a mixture of U(V) and U(VI) species and thus excluding the fact that the examined sample was U_2_O_5_. Compared with the U*4f* spectrum reported for KUO_3_^42^, the XPS shown in the central panel of Fig. 1 displays the same peak shape, except a shift to higher binding energy. The energy shift may reflect the different chemical environment or be due to charge compensation by the flooding gun, needed for bulk samples. The ~8 eV satellite is not affected by this shift, because it depends on the BE difference.

**Figure 2:**
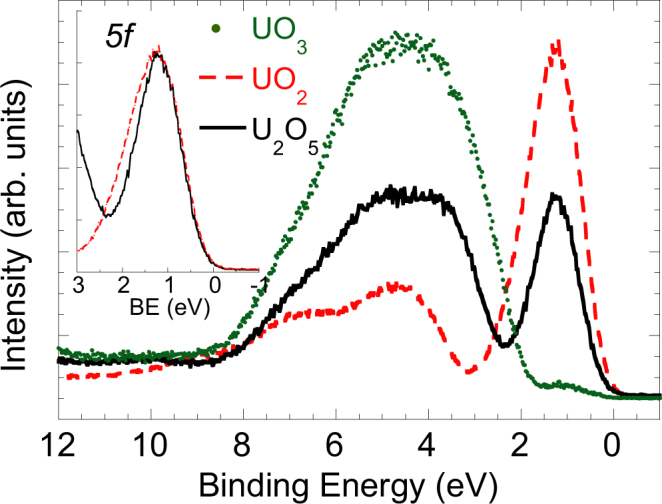
XPS valence band spectra of UO_2_ (red, dashed line), U_2_O_5_ (black, solid line) and UO_3_ (green dots). The inset shows the U5*f* emission for UO_2_ and U_2_O_5_, normalized on the same peak height; the U_2_O_5_ spectra has been shifted to high BE by 0.06 eV to superpose the right flank of the two lines.

Figure 2 shows the valence-band XPS of UO_2_, U_2_O_5_ and UO_3_. Intensities have been normalized to the height of the respective U4*f*_7/2_ peak, in order to compare the relative strength of the spectral features. UO_2_ (red, dashed line) shows an intense, symmetric U*5f* peak at about 1.33(1) eV binding energy and with FWHM = 1.46(2) eV. Between 2 and 8 eV, one can observe the O*2p* band with two prominent features related to the band structure at 4.5 and 7 eV. In UO_3_ (green dots) the 5*f* emission is missing, which is consistent with a 5*f* ^0^ configuration. The O*2p* band is broad and featureless. Both spectra are well known in literature^55^. The valence-band XPS of U_2_O_5_ (black, solid line) has also a 5*f* emission at 1.27(1) eV BE, with intensity equal to about 50% of that observed for UO_2_. This is fully consistent with *f* ^1^ and *f* ^2^ configurations in U_2_O_5_ and UO_2_, respectively. As shown in the inset of Fig. 2, the 5*f* emission in U_2_O_5_ is narrower (FWHM = 1.19(1) eV) than in UO_2_, reflecting expected differences in the final state multiplet structure. This proves that the oxidation of UO_2_ proceeds via U(V) formation and not, as claimed in the past, through direct U(VI) formation: if oxidation of UO_2_ would produce a mixture of U(IV) and U(VI), then the 5*f* signal should decrease in intensity while keeping the same width.

**Figure 3:**
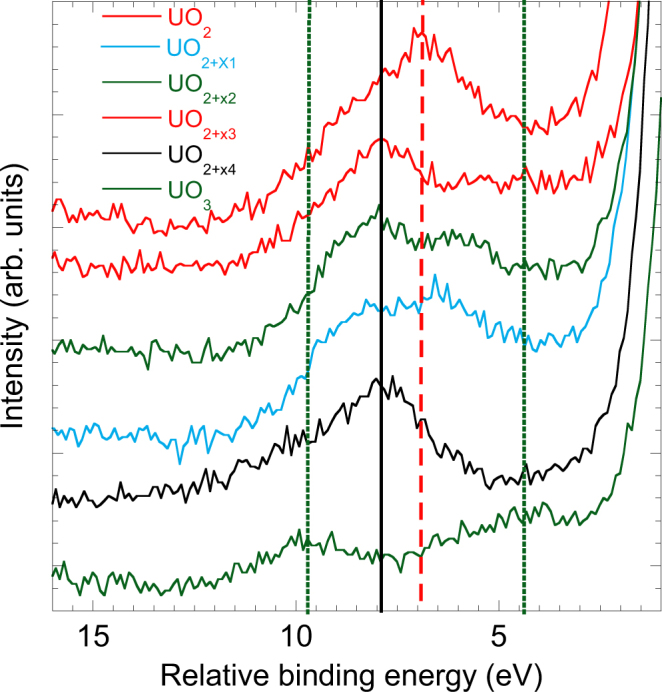
Satellite feature above the U 4*f*_5/2_ XPS emission line recorded for samples with different oxygen-to-metal ratio, increasing from 2 to 3. Individual spectra have been shifted in energy so that the 4*f*_5/2_ peaks for the different compounds are superposed, and vertically for the sake of clarity. Intensities are shown as a function of the binding energy relative to the 4*f*_5/2_ line. Vertical lines indicate the relative binding energy of the satellite characteristic for U(IV) (red, dashed line), U(V) (black, solid line), and U(VI) (green, dotted line) oxidation state.

Alongside the *in-situ* film deposition, we explored the evolution of the oxidation state during the transformation from UO_2_ to UO_3_ by measuring PE spectra for thin films of 2 to 50 layers thickness in a wide range of the O/U ratio. The oxygen content was varied by exposing the films to atomic oxygen for film oxidation and to atomic hydrogen for film reduction. The small thickness of the films and the elevated reaction temperatures allowed preparing homogeneous films with gradually varying oxygen compositions, and to study the evolution of the oxidation state of the uranium ions from IV to VI. Figure 3 shows the satellite peak above the U*4f*_5/2_ line for selected samples with U/O ratio increasing from 1/3 to 1/2. The XPS spectra have been shifted in energy in order to superpose the main emission line from the different compounds and make easier the visual inspection of the spectral changes. By comparing the spectroscopic signature of the three neighbouring oxidation states U(IV), U(V), and U(VI), the results show that pentavalent uranium ions coexist with either tetravalent of hexavalent ones, whereas the simultaneous presence of U(IV) and U(VI) species is not observed at any stoichiometric composition.

## Conclusion

Homogeneous thin films of U_2_O_5_ with thickness corresponding to 20–30 monolayers have been obtained by exposing UO_3_ films to atomic hydrogen. Uranium 4*f* X-ray photoemission spectra show a spin-orbit split doublet characterized by symmetric lines broader than those observed for tetravalent and hexavalent uranium oxides. The U*4f*_7/2_ line is at 380.9(1) eV binding energy and the spin-orbit splitting is 10.8(1) eV. A satellite occurs at a binding energy 7.9(1) eV higher than the main emission peaks. This spectral feature is empirically used as a marker for establishing the oxidation state of the uranium atoms. The satellite energy and intensity is intermediate to those observed for UO_2_ and UO_3_. Whereas a complete understanding of the origin of this spectral feature is not yet available, it has been suggested that its intensity is affected by covalent mixing of high lying U and O orbitals, by the U-O distance, and by the geometry of the crystallographic lattice^56^. The results obtained therefore provide important information for understanding the physical origin of the satellite feature in the U*4f* XPS and for benchmarking theoretical models. Recent computational studies^57^ have demonstrated that first principle calculations combining the local density approximation of the density functional theory (LDA-DFT) with the dynamical mean field theory (DMFT) method are able to provide an accurate description of the electronic structure in early actinide dioxides. This kind of calculations, combined with high-resolution XPS data, affords a quantitative evaluation of the covalency between uranium 5*f* and oxygen 2*p* states which, in turn, results in an enhancement of the 5*f* occupation number^58^. Similar hybridization effects are expected to occur also in U_2_O_5_ and a departure from an integer 5*f* ^1^ occupation cannot be excluded. Future studies will address this important issue.

## Methods

### Samples preparation

Thin films of uranium oxide UO_2_ and UO_2+x_ were prepared *in-situ* by direct current (DC) sputtering from a uranium metal target in a gas mixture of Ar (6N) and O_2_ (6N). The uranium target voltage was fixed at −700 V. The thin films were deposited at room temperature on single-crystal silicon wafer (100-oriented) and polycrystalline Au substrates, cleaned by Ar ion sputtering (4 keV) for 10 min and subsequently annealed at 773 K for 5 min. The deposition time varied between 100 and 300 seconds. The plasma in the diode source was maintained by injection of electrons of 25–50 eV energy (triode setup), allowing working at low Ar pressure in absence of stabilizing magnetic fields.

The oxygen concentration in the films was varied by maintaining Ar pressure at 5 × 10^−3^ mbar and adjusting the O_2_ partial pressure (10^−6^ mbar to 5 × 10^−6^ mbar). UO_3_ films were produced by further oxidizing UO_2+x_ films with atomic oxygen, produced by an electron cyclotron resonance (ECR) Plasma Source Gen I from Tectra GmbH, Frankfurt/M. The atom flux is specified to >10^16^ atoms/cm^2^/s, corresponding to an exposure up to 20 s of roughly 10 Langmuirs (i.e. 1.33 × 10^−3^ Pa s). U_2_O_5_ was obtained by reducing UO_3_ by exposing it up to 60 s to atomic hydrogen, also produced in the ECR source.

### Samples characterization

High resolution X-ray photoelectron spectroscopy (XPS) measurements were performed using a Phoibos 150 hemispherical analyser. Al Kα (E = 1486.6 eV) radiation was produced by a XRC-1000 micro-focus source, equipped with a monochromator and operating at 120 W. The background pressure in the analysis chamber was 2 × 10^−10^ mbar. The spectrometers were calibrated by using the *4f*_7/2_ line of Au metal to give a value at 83.9(1) eV binding energy (BE) and the *2p*_3/2_ line of Cu metal at 932.7(1) eV BE for XPS. Photoemission spectra were taken at room temperature. Data analyses were performed using CasaXPS and Origin software packages.

The datasets generated and/or analyzed during the current study are available from the corresponding author on reasonable request.

## Electronic supplementary material

Supplementary Figure 4

Supplementary Figure 5

Supplementary information
