# Supplementary figures and images for: Direct observation of pure pentavalent uranium in U2O5 thin films by high resolution photoemission spectroscopy
Source: Sci Rep. 2018 May 29;8:8306. doi: 10.1038/s41598-018-26594-z (PMC5974404; doi:10.1038/s41598-018-26594-z)

Intensity (arbitrary unit)

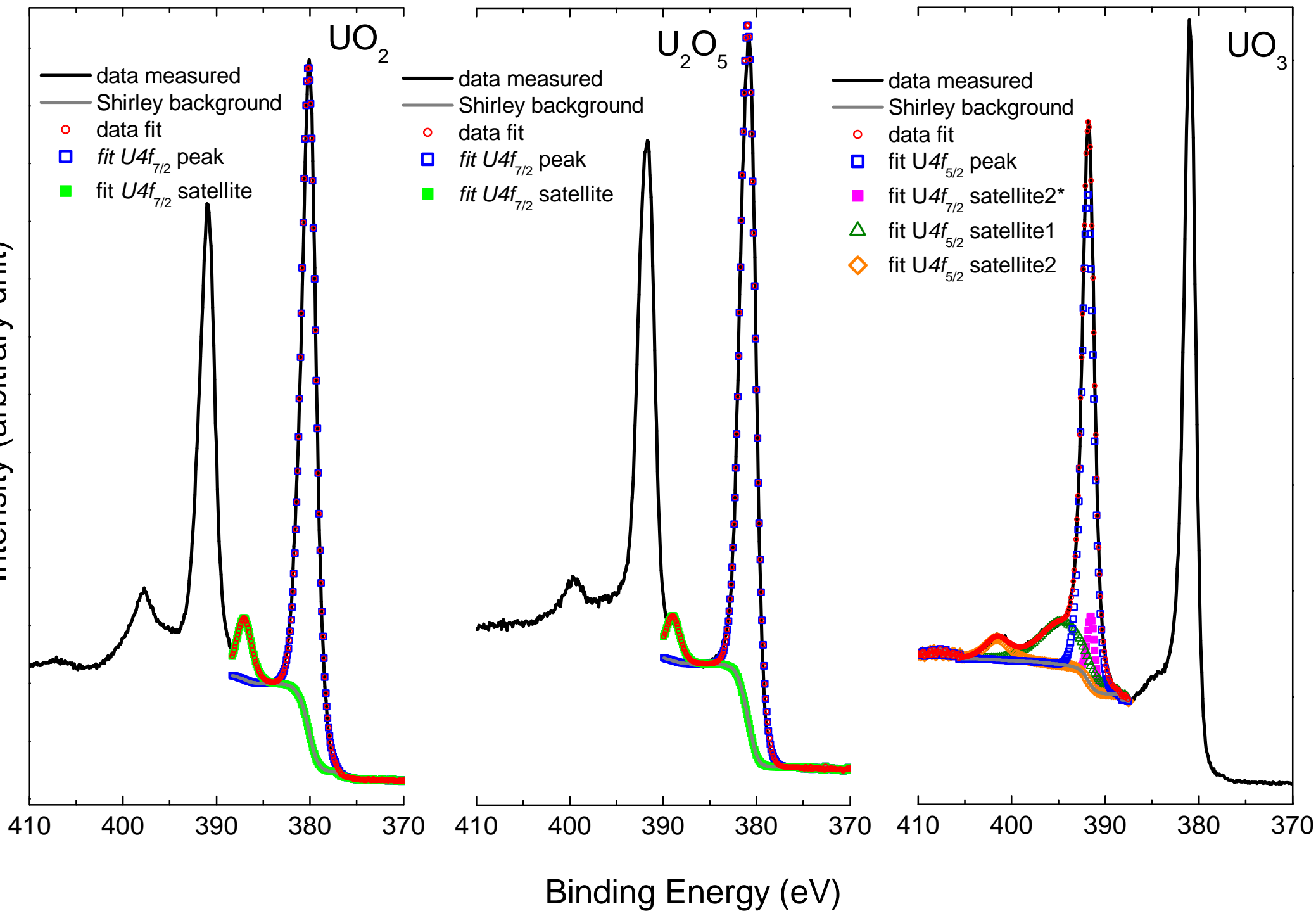

Supplement: Supplementary file 1 — Supplementary Figure 4 [file 41598_2018_26594_MOESM1_ESM.pdf]

Intensity (arb. unit)

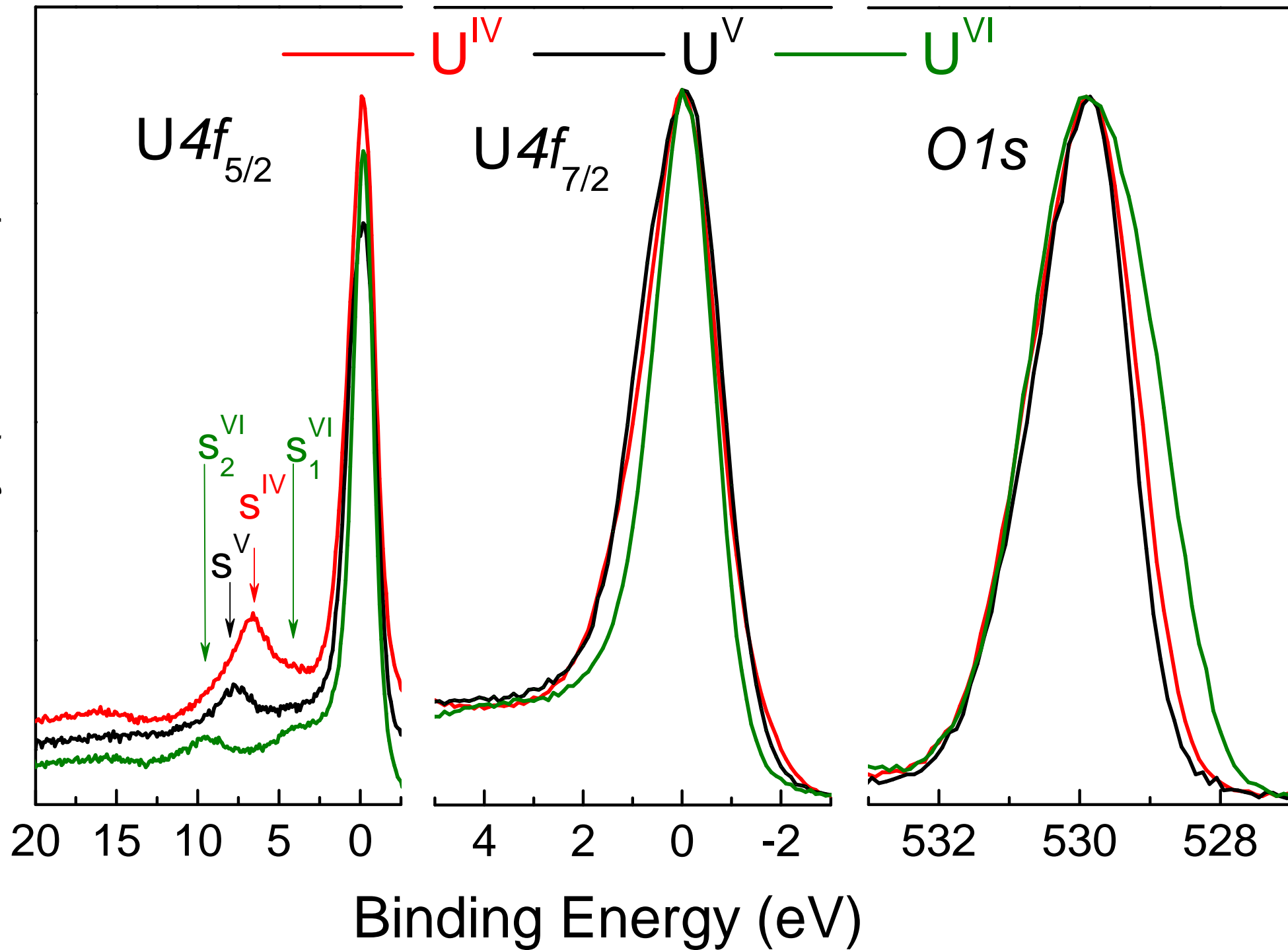

Supplement: Supplementary file 2 — Supplementary Figure 5 [file 41598_2018_26594_MOESM2_ESM.pdf]
