# Supplementary material for: Direct observation of pure pentavalent uranium in U2O5 thin films by high resolution photoemission spectroscopy
Source: Sci Rep. 2018 May 29;8:8306. doi: 10.1038/s41598-018-26594-z (PMC5974404; doi:10.1038/s41598-018-26594-z)
Supplement: Supplementary file 3 — Supplementary information [file 41598_2018_26594_MOESM3_ESM.pdf]

Submission of revised manuscript SREP-18-01441-T to Scientific Reports

# "Direct observation of pure pentavalent uranium in $U_2O_5$ thin films by high resolution photoemission spectroscopy"

T. Gouder, R. Eloirdi, R. Caciuffo

European Commission, Joint Research Centre
Postfach 2340, 76125 Karlsruhe/Germany

Supplementary information_ legends and tables of

Figure 4. Peak fitting of Uranium 4f core level Photoemission Spectra recorded for a) U(IV) in $UO_2$ _ $U4f_{7/2}$ peak and satellite b) U(V) in $U_2O_5$ _ $U4f_{7/2}$ peak and satellite c) U(VI) in $UO_3$ _ $U4f_{5/2}$ peak and satellites s1, s2 (s2* satellite of $U4f_{7/2}$) with CasaXPS programme.

Figure 5. Uranium 4f and oxygen 1s core level Photoemission Spectra recorded for $UO_2$, $U_2O_5$ and $UO_3$.

Table 1. Corresponding CasaXPS parameters to fit $U4f_{7/2}$ peak of $UO_2$ (fig.4a)

| $UO_2$ | $U4f_{7/2}$ | |
|---|---|---|
| | Main peak | satellite 1 |
| Background | Shirley | |
| Profile peak | GL(60) | |
| FWHM | 1.8(1) | 2.0(1) |
| Position | 380.1(1) | 387.1(1) |

Table 2. Corresponding CasaXPS parameters to fit $U4f_{7/2}$ peak of $U_2O_5$ (fig.4b)

| $U_2O_5$ | $U4f_{7/2}$ | |
|---|---|---|
| | Main peak | satellite 1 |
| Background | Shirley | |
| Profile peak | GL(50) | |
| FWHM | 1.8(1) | 1.8(1) |
| Position | 380.9(1) | 388.9(1) |

Table 3. Corresponding CasaXPS parameters to fit $U4f_{5/2}$ peak of $UO_3$ (fig.4c)

| $UO_3$ | $U4f_{5/2}$ | | |
|---|---|---|---|
| | Main peak | satellite 1 | satellite 2 |
| Background | Shirley | | |
| Profile peak | GL(60) | | |
| FWHM | 1.4(1) | 5.6(1) | 3.0(1) |
| Position | 391.8(1) | 395.8(1) | 401.5(1) |
